# Whole-brain imaging of freely-moving zebrafish

**DOI:** 10.3389/fnins.2023.1127574

**Published:** 2023-04-17

**Authors:** Hamid Hasani, Jipeng Sun, Shuyu I. Zhu, Qiangzhou Rong, Florian Willomitzer, Rumelo Amor, Gail McConnell, Oliver Cossairt, Geoffrey J. Goodhill

**Affiliations:** ^1^Department of Electrical and Computer Engineering, Northwestern University, Evanston, IL, United States; ^2^Department of Computer Science, Northwestern University, Evanston, IL, United States; ^3^Departments of Developmental Biology and Neuroscience, Washington University in St. Louis, St. Louis, MO, United States; ^4^Wyant College of Optical Sciences, University of Arizona, Tucson, AZ, United States; ^5^Queensland Brain Institute, The University of Queensland, Brisbane, QLD, Australia; ^6^Centre for Biophotonics, Strathclyde Institute of Pharmacy and Biomedical Sciences, University of Strathclyde, Glasgow, United Kingdom

**Keywords:** light-field microscopy, behavior, calcium imaging, image deconvolution, zebrafish

## Abstract

One of the holy grails of neuroscience is to record the activity of every neuron in the brain while an animal moves freely and performs complex behavioral tasks. While important steps forward have been taken recently in large-scale neural recording in rodent models, single neuron resolution across the entire mammalian brain remains elusive. In contrast the larval zebrafish offers great promise in this regard. Zebrafish are a vertebrate model with substantial homology to the mammalian brain, but their transparency allows whole-brain recordings of genetically-encoded fluorescent indicators at single-neuron resolution using optical microscopy techniques. Furthermore zebrafish begin to show a complex repertoire of natural behavior from an early age, including hunting small, fast-moving prey using visual cues. Until recently work to address the neural bases of these behaviors mostly relied on assays where the fish was immobilized under the microscope objective, and stimuli such as prey were presented virtually. However significant progress has recently been made in developing brain imaging techniques for zebrafish which are not immobilized. Here we discuss recent advances, focusing particularly on techniques based on light-field microscopy. We also draw attention to several important outstanding issues which remain to be addressed to increase the ecological validity of the results obtained.

## 1. Introduction

To fully understand how neural circuits process sensory input and generate complex behaviors it is critical to be able to record simultaneously from large numbers of neurons while the animal is behaving as naturally as possible. Larval zebrafish are transparent and, uniquely amongst vertebrate model organisms used in neuroscience, allow the potential for neural activity in all ~100,000 neurons in the brain to be optically imaged simultaneously. Zebrafish have a strong genetic and physiological homology to mammals, and they have analogous social and cognitive behavioral processes to those seen in rodents and humans (Stewart et al., 2014). They are in widespread use as an animal model for a range of human neurological disorders, including schizophrenia (Thyme et al., 2019) and autism spectrum disorders (Stewart et al., 2014; Meshalkina et al., 2018), where changes can already be seen at larval stages (Thyme et al., 2019; Constantin et al., 2020; Marquez-Legorreta et al., 2022; Zhu et al., 2023). Larval zebrafish engage in visually-driven hunting behaviors which involve complex sensorimotor transformations (Bianco and Engert, 2015; Bollmann, 2019; Zhu and Goodhill, 2023). Considerable insight has been gained into the neural circuits underlying hunting from restrained preparations. However, recent pioneering technologies have opened the door to whole-brain imaging in zebrafish which are relatively freely-moving. Such imaging involves solving a number of challenging problems, particularly due to the high accelerations and speeds larval zebrafish can achieve. Here we review work on these problems so far, discuss their strengths and weaknesses, and consider the potential for future improvements. We primarily refer to imaging neurons, but acknowledge that glia also play critical roles in information processing [e.g., Mu et al. (2019)]. First we introduce the zebrafish model, how neural activity can be imaged in this system, and the conventional imaging technologies that have been used for imaging of restrained zebrafish larvae. We then discuss methods that have recently been proposed for brain imaging in freely-moving zebrafish, particularly emphasizing approaches based on light-field microscopy.

## 2. The zebrafish model system

### 2.1. Behavior

Zebrafish develop rapidly (Kita et al., 2015), and by 5 days post-fertilization (dpf) can already hunt fast-moving prey such as *Paramecia* using only visual cues (Bianco et al., 2011; Muto and Kawakami, 2013; Patterson et al., 2013; Bianco and Engert, 2015; Bollmann, 2019). Social behavior begins to develop around 15 dpf (Larsch and Baier, 2018; Kappel et al., 2022). These naturally-occurring behaviors provide an attractive model system for understanding the neural circuits involved in sensory processing and sensory-motor transformations (e.g. Barker and Baier, 2015; Förster et al., 2020; Zhu and Goodhill, 2023). Larval zebrafish move in a series of discrete bouts, lasting 100-200 ms at a frequency averaging about 1 Hz (Johnson et al., 2020; Mearns et al., 2020). During these bouts larvae can achieve speeds and accelerations of 100 mm/s and 15,000 mm/s^2^ respectively, and angular speeds and accelerations of 600 °/s and 1000 °/s^2^ respectively (our unpublished data). Bouts can be grouped into 7–13 different classes (Marques et al., 2018; Mearns et al., 2020). Bouts involve complex tail movements with a frequency up to about 80 Hz. Accurately capturing tail shape during bouts requires imaging at several hundred Hz, followed by sophisticated image-processing techniques to extract the midline (Avitan et al., 2020; Mearns et al., 2020). However to reliably track only the position of the fish (e.g., midpoint between the two eyes), imaging at a few tens of Hz and relatively simple image processing methods are sufficient.

**Figure 1:**
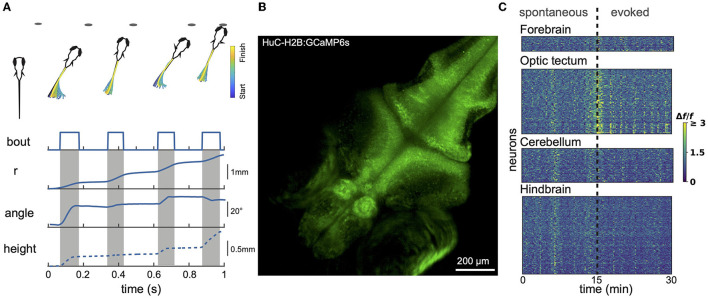
Larval zebrafish behavior and neural activity imaging. **(A)** Example hunting sequence. Also shown are total displacement (r), heading angle and z-depth (height). r and angle data were recorded in 2D for the hunting event shown above; height data was estimated based on data from similar events recorded in 3D by others (Bolton et al., 2019). **(B)** Example maximum projection image of volumetric 1-photon light sheet recording of a GCaMP6s 9 dpf fish imaged at 1 Hz. 15 planes were captured spanning a total z-range of 150 μm. **(C)** Calcium signals measured using light sheet recording during spontaneous activity and in response to presentation of prey-like stimuli. Different brain regions captured simultaneously show both synchronized and region-specific activity patterns.

Larvael zebrafish alternate between exploratory movements (eyes unconverged) and hunting sequences (eyes converged). Eye convergence creates a small binocular zone about 0.5 mm directly in front of the fish that is likely useful for calibrating the final strike (Bianco et al., 2011). Figure 1A shows a stereotypical hunting sequence. Driven by visual cues, the fish makes a rapid series of orienting turns and then attacks the prey either by suction or a rapid strike movement (Mearns et al., 2020). This behavior improves over development (Avitan et al., 2020; Lagogiannis et al., 2020). Zebrafish larvae readily perform these behaviors in small (e.g., 20 mm) culture dishes. While prey-hunting has so far mostly been characterized just in xy space, recent data demonstrate an important role for movements along the z axis (Bolton et al., 2019; Mearns et al., 2020). Mearns et al. (2020) showed that, when hunting, larval zebrafish adopt an average pitch of 12° (corresponding to a vertical displacement of about 1 mm between head and tail), and prefer to attack their prey at an upwards angle. Prey detection generally occurs at an angle of 35–40° to the fish midline, after which the fish makes an initial orienting turn (Budick and O'Malley, 2000; Patterson et al., 2013; Bolton et al., 2019; Avitan et al., 2020).

### 2.2. Brain imaging

#### 2.2.1. Reporting neural activity

Brain activity can be quantified either directly, through measuring the generation of action potentials, or indirectly, through measuring changes in cytosolic protein products or changes in calcium level post neuron firing. Transcription of *c-fos* in neurons (Curran and Teich, 1982) is rapidly increased when neurons are activated (Krukoff, 1999). This allows *c-fos* to be used as an indicator of neural activity in response to stimulation, and thus the detection of brain regions that are involved in specific neural processing tasks in zebrafish (Decarvalho et al., 2013; Kappel et al., 2022). This can be done by measuring either *c-fos* protein translation via immunohistochemistry or mRNA expression using *in situ* hybridization.

Intracellular calcium levels can also be used as a proxy for neural activity. During an action potential the opening of voltage-gated calcium channels causes a 10-fold increase in free calcium within 1 ms (Luo et al., 2018). The calcium influx then leads to phosphorylation of mitogen-activated protein kinase [MAP, also known as pERK, (Rosen et al., 1994)]. Immunohistochemical detection of pERK can therefore be used as a measure for neural activity (Randlett et al., 2015; Corradi et al., 2022). Alternatively, sensor protein-based detection of calcium can also be used which enables live visualization of neural activity (Whitaker, 2010). The most commonly used calcium indicators are the GCaMP family (Grienberger and Konnerth, 2012). These proteins consist of three major components, the circularly permuted green fluoresent protein (GFP), the calcium-binding protein calmodulin, and a calcium-bounded-calmodulin-interacting M13 peptide (Nakai et al., 2001; Akerboom et al., 2012). The binding of calcium to calmodulin causes a conformational change in the GCaMP and releases the GFP from the protonated state, increasing the fluorescence level. Several generations of improvement have been made to increase the sensitivity, speed and signal of the GCaMPs (Nakai et al., 2001; Tian et al., 2009; Chen et al., 2013). Zebrafish can avoid some of the challenges of GCaMPs in rodent models, such as uneven expression (Sakamoto et al., 2022). In zebrafish pan-neuronal expression can be achieved via neuroD or HuC promotors, which are critical gene expressed during neurogenesis (Park et al., 2000; Rupprecht et al., 2016; Oldfield et al., 2020). GCaMPs can be targeted to the nucleus by fusing them to human histone H2B protein (Kanda et al., 1998; Vladimirov et al., 2014). In zebrafish neurons the nucleus occupies almost the entire soma, making this a very effective technique as it facilitates image segmentation compared to targeting GCaMPs to the cytosol. GCaMPs have helped reveal large-scale population response patterns in zebrafish (Del Bene et al., 2010; Muto et al., 2011; Vladimirov et al., 2014). Ratiometric indicators can also be used for visualizing neural activity via fluorescence resonance energy transfer (FRET). Ratiometric indicators such as *cameleon* consist of two fluorophores. Binding of calcium activates the emission from one of these via FRET, resulting in changes in the fluorescence ratio between the two fluorophores, which can then be used as a proxy of neural activity (Mank and Griesbeck, 2008; Kettunen, 2012). Ratiometric indicators are particularly useful for measuring basal-level activities and have been used in functional mapping and connectivity mapping studies in zebrafish (Li et al., 2005; Tao et al., 2011).

Although GCaMPs are very powerful they are relatively slow, due to the intrinsic dynamics of calcium in neurons and the dynamics of the indicators themselves (Ali and Kwan, 2020). A more direct measure of neural activity is to visualize changes in the membrane potential via genetically-encoded voltage indicators (Xu et al., 2017; Luo et al., 2018; Abdelfattah et al., 2019), which come in two main types. The first type utilizes the voltage-sensing domains of voltage-sensitive phosphatase or ion channels in the membrane. By fusing GFPs with these voltage-sensing domains, the voltage-induced conformational change results in changes in fluorescence (St-Pierre et al., 2014). The second type takes advantage of microbial rhodopsins which produce fluorescence responses to voltages changes (Kralj et al., 2012). Voltage indicators can resolve single action potentials (Kralj et al., 2012; Luo et al., 2018), and combining voltage imaging with cell-type specific markers has helped reveal fine spatial and temporal details of neural activity in zebrafish such as the propagation of motor signals in spinal cord circuits (Böhm et al., 2022). However signals from voltage imaging are generally weaker than those from calcium imaging, and the signal-detection challenge is increased by the high imaging rates necessary to capture the fast temporal dynamics of membrane-voltage fluctuations.

Visualization of neural activity at the synaptic level can be achieved via genetically-encoded neurotransmitter sensors (Wang et al., 2018; Vogt, 2019). Sensing of neurotransmitter release can be achieved either *via* bacterial-derived binding proteins or via G-protein-coupled receptors (GPCRs) with specific selectivity (Marvin et al., 2013; Jing et al., 2018; Marvin et al., 2018; Patriarchi et al., 2018; Sun et al., 2018; Marvin et al., 2019). Visualization of neurotransmitter activity can be achieved by engineering an insertion of fluorescence protein on to these sensor proteins.

Several systems have been developed to allow cell-type specific expression of fluorescence labels including the Gal4 (a yeast transcription factor) and Gal4 upstream activating sequence (gUAS) system (Gal4/gUAS), the tryptophan repressor (utilizing tryptophan biogenesis in *E. coli*) and its UAS system (TrpR/tUAS), and the QF transcription factor (adopted from fungus *Neurospora crassa*) and its UAS system (QF/QUAS). All these systems were developed to create a binary expression system where the fluorescence reporter protein expression is regulated by cell-type specific promoters (Asakawa and Kawakami, 2008; Suli et al., 2014; Ghosh and Halpern, 2016). Limitations of these systems include DNA methylation over generations which leads to gene silencing in some lines of Gal4/gUAS system, and toxicity when expressed by strong promoters in the TrpR/tUAS system (Suli et al., 2014; Burgess et al., 2020). Nevertheless, these systems have been used widely in zebrafish for targeted expression with calcium indicators (Choi et al., 2021; Barker et al., 2021), voltage indicators (Böhm et al., 2022) and neurotransmitter indicators (Yoshimatsu et al., 2020).

#### 2.2.2. Microscopy methods for head-fixed fish

*nacre* zebrafish (which carry mutations that affect pigment cells) are transparent at larval stages, making them ideal for volumetric optical imaging of brain activity (Huang et al., 2021). *Casper* (White et al., 2008) and *Crystal* (Antinucci and Hindges, 2016) fish maintain this transparency in adulthood. Since up to about 15 dpf zebrafish breathe through their skin, before this age the unparalyzed and unanaesthetized larvae can be easily immobilized in agarose for imaging. Although zebrafish start to hunt live prey at around 5 dpf, they can survive without any external source of food for several days beyond this (Hernandez et al., 2018; Lagogiannis et al., 2020); thus imaging can occur for many hours or even days at a time with no maintenance of the animal required. To allow some degree of behavioral output, the tail can be freed while maintaining the head immobile under the microscope objective. Virtual reality environments can be created and tail movements (or electrode recordings of motorneuron activity) used to drive movement through these environments (Ahrens et al., 2012; Trivedi and Bollmann, 2013; Vladimirov et al., 2014; Torigoe et al., 2021). Alternatively stimuli can be projected onto a screen in front, to the side or beneath the fish. Looming stimuli and also small, moving or stationary prey-like spots can evoke strong neural responses in several brain areas, most notably the optic tectum, and also tail movements (Semmelhack et al., 2014; Bianco and Engert, 2015; Fernandes et al., 2021). In addition, some studies have used head-fixed fish observing free-swimming paramecia (Muto and Kawakami, 2013; Wee et al., 2019; Oldfield et al., 2020).

The principal techniques that have been applied to brain imaging in head-fixed fish are spinning disk, 2-photon, light sheet, and 2-photon light sheet microscopy. The main challenge is to image fast enough so as to capture volumes of all or at least a substantial fraction of the brain at rates exceeding 1 volume/s. The larval zebrafish brain spans dimensions of roughly 450 μ*m* width by 700 μ*m* length by 320 μ*m* height (Svara et al., 2022). “Whole brain” imaging in this context generally means the full extent of x and y but more limited z, which is sufficient to provide complete imaging of most brain regions except the deepest. Sophisticated automated techniques are then required to convert the raw imaging data to df/f activity traces such as CaImAn (Giovannucci et al., 2019) and Suite2P (Pachitariu et al., 2017).

##### 2.2.2.1. 1-photon methods

Spinning disk microscopy has been used to capture single-plane images of optic tectum at 5 Hz (512 × 512 pixels) (Avitan et al., 2016), and single-plane images spanning the whole xy extent of the zebrafish brain at 20–30 Hz (512 × 512 pixels) (Liu and Baraban, 2019), or even up to 100 Hz (Muto et al., 2017). Though spinning disk volumetric imaging has been achieved for *c. elegans* (Nguyen et al., 2016; Venkatachalam et al., 2016), this has not so far been reported for zebrafish. Using light sheet, also known as single plane illumination microscopy (SPIM), close to 1 Hz imaging has been achieved for ~40 planes spanning 800 × 600 × 200 μm^3^, capturing ~80% of the neurons in the brain (Ahrens et al., 2013) (for example see Figures 1B, C). With the use of an electrically tunable lens, the imaging speed was increased to 4 Hz per brain volume (Favre-Bulle et al., 2018). Since the eyes are large they obscure a substantial portion of the brain from laser illumination from the side. To address this, the fish can be illuminated with dual light sheets from both the side and the front, with the latter compensating for most of the brain volume lost from side illumination. However, with both designs, the illumination light sheets block part of the visual field of the fish and can therefore interfere with the presentation of visual stimuli. This problem can be resolved with modifications of the light-path design such as oblique plane microscopy. This single-objective design can achieve imaging speeds of 3.3 Hz over 500 × 300 × 200 μm^3^ using DaXi microscopy (Yang et al., 2022) and speeds up to 25.75 Hz over 392 × 299 × 41 μm^3^ using SCAPE microscopy (Voleti et al., 2019). However a significant limitation of all 1-photon techniques is that scattered light from the excitation laser is visible to the fish. This stimulates the visual system and potentially degrades the fish's ability to see visual stimuli presented by the experimenter.

##### 2.2.2.2. 2-photon methods

2-photon microscopy is particularly useful for live imaging in larval zebrafish since the excitation laser is invisible to the fish, allowing uncorrupted analysis of visual processing. However a significant limitation of point-scanning 2-photon microscopy is speed, since it scans the tissue 1 voxel at a time. With recent implementation of resonant scanning mirrors, imaging speeds have been achieved of 1–1.2 Hz for 9 to 12 planes over 76 μm (Andalman et al., 2019), 2.7 Hz for 10 planes over 150 μm (Burrows et al., 2021), and 9.7 Hz for 5 planes of just the tectum (Sainsbury et al., 2023). In 2-photon light-sheet microscopy the light sheet consists of 2-photon excitation generated by scanning a pulsed infrared laser source (Truong et al., 2011; Wolf et al., 2015). One of the biggest challenges of performing this technique in zebrafish is that the fish's eyes absorb infrared light, so that any exposure of the eyes to the laser kills the fish. Other challenges include the technical complexity of the experimental setup; in addition to ensuring the beam covers the intended brain region to be imaged, dispersion compensation may be required to correct for pulse broadening in order to obtain optimal signal levels.

## 3. Imaging neural circuits in freely moving fish

Using the head-fixed assays described above, impressive progress has been made in deciphering the neural circuits underlying behavior in zebrafish larvae (Lin et al., 2020; Zhu and Goodhill, 2023). However in these paradigms the fish does not receive the visual, proprioceptive, vestibular or gustatory feedback that it would experience during unconstrained movements and prey hunting. There has therefore been much recent interest in developing assays which permit brain imaging in moving fish with high spatial and temporal resolution.

An early pioneering approach used a low-magnification objective lens to image the whole of a small dish, allowing overall patterns of tectal activity in a freely-moving zebrafish to be imaged as it pursued a paramecium (Muto et al., 2013). A subsequent refinement of this approach used a dish of 9 mm diameter and 0.8 mm depth, an objective lens of power 2.5X or 5X, single-plane spinning disk microscopy at a frame rate of 100 Hz, and manual adjustment of the stage to keep the fish within the limited field of view (Muto et al., 2017). High-speed high-resolution volumetric brain imaging requires however an automated tracking system to eliminate relative movement between the fish brain and a high-power microscope objective. This is a more challenging problem than tracking and imaging of freely moving worms (Nguyen et al., 2016; Venkatachalam et al., 2016), since zebrafish move much faster and in 3D (Ehrlich and Schoppik, 2017; Bolton et al., 2019). Indeed none of the above-mentioned imaging techniques for head-fixed fish have so far been successfully adapted to high resolution volumetric imaging of freely-moving fish. Recent work has instead focused on imaging techniques which trade some spatial resolution for increased speed (Cong et al., 2017; Kim et al., 2017; Symvoulidis et al., 2017; Zhang et al., 2021a). We first discuss the behavioral tracking approaches used, and then the brain imaging techniques.

### 3.1. Behavioral tracking

In recent years there have been major advances in markerless tracking of laboratory animals, using tools such as DeepLabCut (Mathis et al., 2018). However zebrafish offer distinct challenges in this regard compared to e.g., rodents. Firstly the zebrafish tail is difficult to track in terms of keypoints. Secondly and more importantly, the very high speeds and accelerations zebrafish larvae can achieve presents formidable challenges for real-time tracking to retain the fish within the field of view (FoV) of the microscope. These challenges include real-time video processing speed, the accuracy of the zebrafish movement prediction algorithm, z axis tracking ability, and integration with the motor hardware.

**Figure 2:**
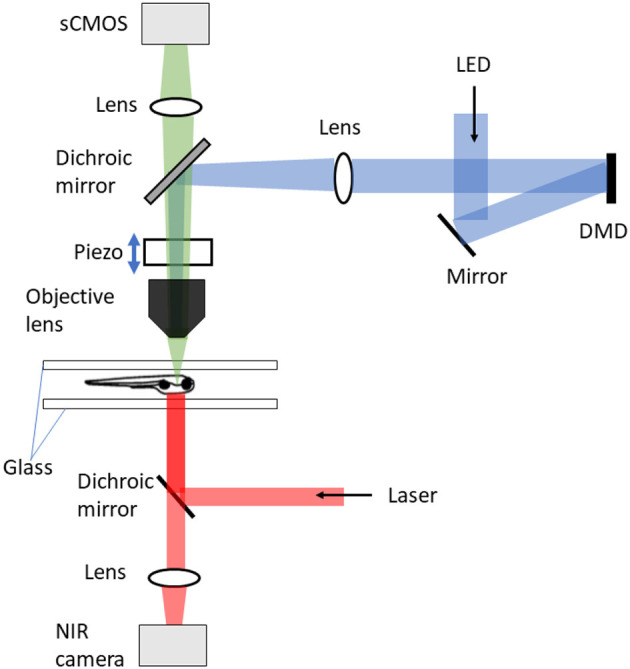
DIFF setup with tracking microscopy (Kim et al., 2017). The tracking microscope consists of three major components: DIFF imaging system, motion cancelation, and stimulus delivery. The DIFF imaging system is illuminated by an LED light source. After passing through the tapered glass homogenizer and magnified by a 4f relay (not shown), the light reflects off a fold mirror and illuminates a digital micro-mirror (DMD) device. The DMD ace is imaged to the focal plane of the objective lens. In the fluorescence detection path, the sample is imaged to a global shutter sCMOS camera after passing through the objective lens, a dichroic mirror and an imaging lens. The motion cancelation system includes a high speed motorized xy stage (not shown), an objective-coupled piezo z stage, a behavioral arena and an infrared imaging system for tracking fish position and heading. The infrared system is also used to generate thermal gradients.

Kim et al. (2017), constrained the fish within a water depth of 750 μm between two sheets of glass, in an arena 50 mm in diameter. The shallow depth was required because of the limited maximum distance of travel (400 μm) of the piezo driver of the microscope objective (Figure 2). The fish xy position was tracked using a near-infrared camera at 250 Hz, and this signal fed back to control the xy position of the stage. This required a control system taking into account both the fish's expected movement and a model of the stage itself. The system was developed based on model predictive control theory (García et al., 1989) and consisted of three components: a next-seven time step (+4 ms to +28 ms) fish trajectory extrapolation function that took into account the fish position, heading, and velocity over the previous six time steps (–20 ms to 0 ms); a stage-motion predictive model; and an online solver to select the optimal stage input to minimize the resulting tracking error. This was implemented in real time using dedicated GPUs, and on average enabled a return to within 100 μm of the center of the FOV within 85 ms with a mean overall tracking error of 44 μm. The head xy position was identified by processing pixel-stream data from the lateral tracking camera with a custom FPGA system. The error signal between the actual head position and the prediction was fed into a proportional-integral-derivative control model to generate stage movements; the tracking accuracy was not explicitly reported.

The methods of both Cong et al. (2017) and Kim et al. (2017) are based on cancelation of fish movement by translation of the dish containing the fish, which disturbs the fish's normal behavior (Kim et al., 2017). To avoid this, Symvoulidis et al. (2017) developed a static microscope system with galvanometric mirrors to image neurobehavioral dynamics in freely behaving fish, which they termed NeuBtracker. The system had two imaging input channels: a 1 × large FoV static infrared channel for fish tracking, and a channel to provide detailed fluorescent images by moving galvanometric mirrors to the tracked fish position. A speeded-up robust feature (SURF)-based offline feature matching algorithm (Bay et al., 2008) was used to censor low-quality frames. The tracking error distance reached a peak of about 190 μm when the fish speed was around 30 mm/s.

### 3.2. Optical sectioning using DIFF microscopy

In combination with the tracking system described above, Kim et al. (2017) achieved volumetric brain imaging of freely-behaving zebrafish using differential illumination focal filtering (DIFF) microscopy (Figure 2), an illumination strategy which is a variation of HiLo (high and low resolution fusion) microscopy (Lim et al., 2008). This method uses two non-uniform and complementary structured grating images to illuminate the sample. The final fused image contains all low and high spatial frequencies within the microscope passband. The focused high spatial frequency components are extracted by high-pass filtering the image sum of the two complementary images. To extract the low frequency components, the difference image between the two images is calculated and low-pass filtered. The low- and high- frequency images are then fused to produce the final full-resolution image. This technique removes the background defocus and provides high signal contrast volumetric imaging. Using this Kim et al. (2017) imaged 50 planes per brain volume at 2 Hz per volume of spontaneous swimming and prey capture using an sCMOS camera. To enable image registration, an initial reference volume was created during a period when the fish was stationary. Correction for the small amount of movement allowed in the z axis was achieved by a piezo-coupled objective. As this swept up and down each plane was matched in real time to a plane in the reference volume, thus providing an offset appropriate for centering the brain that was added to the next sweep.

Using this imaging approach, Marques et al. (2020) discovered that zebrafish larvae spontaneously alternate between two persistent internal states of exploration and exploitation during foraging for live prey. The dorsal raphe appeared to be responsible for driving the state transition. Dorsal raphe neurons were activated shortly before state transition and the activity of these neurons was positively correlated with exploitation-state neurons, such as a cluster of Vglut2 neurons in the cerebellum that drove eye convergence. Dorsal raphe neurons were negatively correlated with exploration state neurons such as a Gad1b cluster in the hindbrain whose activity is related to routine turns.

### 3.3. Light-field microscopy (LFM)

An alternative approach to volumetric imaging is Light Field Microscopy (Levoy et al., 2006), which is a family of optical designs that utilize a microlens array (MLA) to map a 4D dataset of scattered or emitted light rays emerging from the sample, incident on the microscope objective, and then detected with a 2D focal plane array The 4D dataset includes a radiance map for every direction (2D: angle x, angle y) and origin of axis (another 2D: x, y); the third spatial dimension can be ignored due to the absence of occluders. A captured 4D LFM dataset can be processed to manipulate the depth of field or perspective in post processing, which makes it possible to compute a 3D focal stack in software after capture (Broxton et al., 2013). A major advantage of LFM over conventional scan-based imaging is that the imaging speed in LFM is in principle limited only by the acquisition rate of the camera. This is because the light field is acquired at a single instant in time or snapshot, i.e. a single exposure of the camera sensor produces a single light field which can be used to reconstruct the entire 3D volume. LFM however comes at a cost of reduced spatial resolution, reconstruction artifacts and high computational load. Reconstruction artifacts are introduced due to the interference of the background noise between out-of-focus and in-focus light resulting in signal-to-noise ratio (SNR) degradation. In a conventional microscope the resolution is given by λ/(2NA) where NA is the numerical aperture. However, LFM systems utilize an MLA consisting of microlens apertures with smaller NA, decreasing the diffraction-limited resolution (Levoy et al., 2006, 2009; Broxton et al., 2013). Because the collection of microlenses act as a single aperture for microscope images, it is possible to recover some of this resolution loss using deconvolution (see later). Nonetheless, the currently achievable lateral spatial resolution of LFM is about 3 μ*m* compared to about 0.5 μ*m* for light-sheet and 2-photon imaging. To further improve the spatial resolution of LFM, given that the wavelength of the emitted light remains constant, the NA can be increased. This improves both lateral and axial spatial resolution since they are inversely proportional to NA. However, the axial range (volume coverage) decreases since the depth of focus is inversely proportional to NA and the overall FoV of the system is not affected. Higher NA also increases the imaging speed as the light gathering capability of the system increases (Zhang et al., 2021b).

#### 3.3.1. Conventional LFM

**Figure 3:**
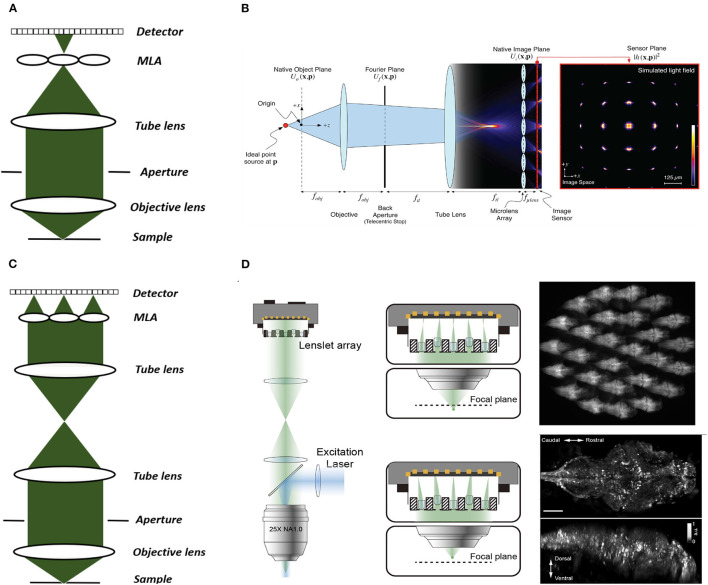
Conventional and Fourier LFM. **(A)** Conventional LFM: The MLA is placed at the native image plane, which is conjugated to the native object plane. Each sub-image records the rays emitted from the conjugated point on the object plane at different angles within the numerical aperture of the objective lens. **(B)** Schematic of wave optics model based on the LFM optical path. The microlens array at the native image plane is modeled as a tiled phase mask operating on this wavefront, which is then propagated to the camera sensor. The x-y cross-section on the far right shows the simulated light field generated at the sensor plane (Broxton et al., 2013). **(C)** Fourier LFM: The MLA is placed at the Fourier plane to focus the incident light onto the camera plane. Each sub-image is a focused sample image observed from that lenslet's perspective. **(D)** Schematic of XLFM. The MLA (lenslet array) position is conjugated to the rear pupil plane of the imaging objective. Point sources at two different depths form sharp images on the imaging sensor, through two different groups of microlenses, with positional information reconstructed from these distinct patterns (reproduced from Cong et al., 2017). **(B)** reproduced with permission from Broxton et al. (2013) ©The Optical Society.

Two main LFM modalities are known as conventional and Fourier LFM. In conventional LFM the MLA is placed in the native image plane and the camera is placed behind the MLA at a distance of one microlens focal length (Figures 3A, B). Here each pixel captures a unique ray, i.e., view, emitted at a specific angle within the numerical aperture of the microscope objective. Hence, the angular resolution is equal to the number of pixels behind each microlens. The lateral resolution is equal to the microlens pitch divided by the objective magnification (Broxton et al., 2013). This design is computationally expensive due to the use of spatially varying point spread functions (PSFs). Placing the MLA in the imaging plane also produces more reconstruction artifacts near the center of the imaging volume leading to loss of resolution in the center.

Conventional LFM has only recently been applied within neuroscience, and has enabled simultaneous functional imaging of neuronal activity at single-cell resolution in an entire *Caenorhabditis elegans* nervous system, the whole larval zebrafish brain (Prevedel et al., 2014; Andalman et al., 2019), and large brain volumes in mice *in vivo* during behavior (Grosenick et al., 2017). In immobilized zebrafish, conventional LFM has achieved a spatial resolution of 3.6 × 3.6 × 5.0 μ*m*^3^ across a reconstruction volume of 700 × 800 × 500 μ*m*^3^ at a volume imaging rate of 5 Hz (Andalman et al., 2019).

#### 3.3.2. Fourier LFM

In Fourier LFM the MLA is placed in the Fourier plane, i.e. the back aperture of the microscope objective, rather than the imaging plane itself (Figure 3C). This is done by transforming the image formed at the native image plane to the back focal plane using a Fourier lens, with the camera at the back focal plane of the MLA (Guo et al., 2019). This design allows a 2D spatially-invariant PSF to be defined, which facilitates simpler and more efficient deconvolution algorithms. It also reduces the reconstruction artifacts near the imaging plane as the MLA is placed away from the imaging plane.

Cong et al. (2017) extended the FoV further by using a customized MLA, which they termed eXtended LFM or XLFM (Figure 3D). The customized MLA was divided into 2 groups with an axial displacement between them to extend the range of depth. Using this technique Cong et al. (2017) achieved a maximum resolution of 3.4 μm by 3.4 μm by 5 μm and reconstructed a lateral extent of 800 μm and an axial extent of 400 μm in freely-moving zebrafish. Fluorescence was excited using 200 μs pulses from an LED, and the system was capable of a volume imaging rate of 77 Hz. Using XLFM as the hardware, Yoon et al. (2020) further improved the XLFM reconstruction performance by increasing the SNR from 9 to 74 at volume imaging rates of up to 50Hz.

#### 3.3.3. Deconvolution algorithms for LFM

Unlike most scanning-based volumetric microscopy methods, where the volumetric reconstruction process is essentially a stacking operation of the deconvoled 2D slice images, LFM systems rely heavily on computational processing to recover volume information. Since voxels at different depths overlap on the camera sensor, 3D deconvolution algorithms are required to infer volumetric information from the light field measurement. The goal of a LFM deconvolution algorithm is to estimate the volumetric light radiant distribution ĝ of the target sample *g* that was most likely to have generated the recorded light field image *f* on the camera sensor. Assuming a linear image model *H*, the deconvolution can be expressed concisely as a matrix inversion problem:


$$ĝ=H^{-1}f$$

Here *H* can be composed by measuring or simulating the PSFs of the system. If the PSFs are shift-invariant at the same axial depth, the matrix multiplication operation can be reduced to a convolution operation. This assumption holds for Fourier LFM systems due to the consistently-aliased spatial and angular information in the Fourier domain (Guo et al., 2019). Deconvolution algorithms can be divided into model-driven and data-driven methods depending on whether the deconvolution process requires a training dataset.

Model-driven methods focus on modeling the physics of image formation instead of relying on *a-priori* knowledge of the light field image dataset *f* distribution. Here the forward model *H* is the PSF measurement matrix, and the deconvolution equation above represents a non-blind deconvolution problem. To store and apply the large *H* matrix, the repeating patterns and the sparsity of the PSFs structure are usually exploited to reduce the cost of the inversion problem.

**Figure 4:**
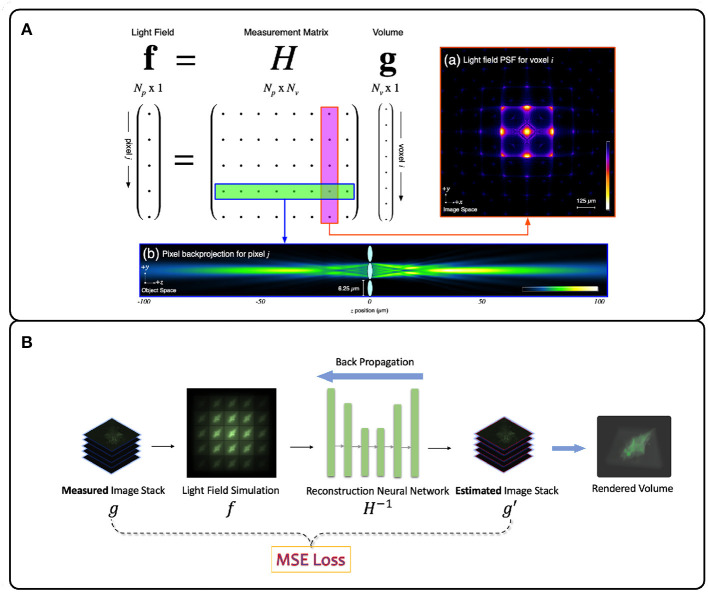
Illustration of deconvolution algorithms for LFM. **(A)** Model-driven methods explicitly form the optical transfer matrix *H* from PSFs and inversely solve the volume vector *g* from the light field measurement *f*. Due to the large size of the matrix *H*, iterative methods such as Richardson-Lucy (Richardson, 1972; Lucy, 1974) are usually adopted (Broxton et al., 2013). **(B)** Data-driven methods implicitly model the measurement matrix *H* by training a neural network to learn the mapping relationship between the light field images and the target volumes from the large light field-volume pairs dataset. The mean-square error (MSE) is usually adopted to provide the loss between the predicted volume and the ground truth volume. The loss then backpropagates to update the neural network weights during the training stage. **(A)** adapted with permission from Broxton et al. (2013) ©The Optical Society.

The Richardson-Lucy (RL) deconvolution algorithm applied to LFM reconstructs the 3D volume by maximizing the Poisson likelihood of the light-field image given an estimate of the 3D volume (Richardson, 1972; Lucy, 1974). The RL deconvolution algorithm and its variants take the PSF stack and LFM image as input and are thus non-blind. The algorithm relies on gradient descent to estimate the most likely 3D volume that produced an observed LFM image (Figure 4A). Due to their robustness and wide implementation support, they are the most popular methods for LFM applied to zebrafish brain imaging (Broxton et al., 2013; Cohen et al., 2014; Prevedel et al., 2014; Perez et al., 2015; Cong et al., 2017; Nöbauer et al., 2017; Taylor et al., 2018; Truong et al., 2020; Zhang et al., 2021a). However, since the iteration process is time-consuming, RL methods are not useful for real-time and high-throughput LFM use cases.

The sparsity of neuronal spiking can be exploited to increase deconvolution performance. For instance, Pégard et al. (2016) and Yoon et al. (2020) demonstrated enhanced resolution by minimizing the error between simulated LFM images of sparse neuronal spiking and observed LFM images using an accelerated proximal gradient algorithm. In Yoon et al. (2020), the authors first extracted the sparse neuron positions with the alternating direction method of multipliers (Candes et al., 2009) and then used a forward model to simulate LFM images. By applying Richardson-Lucy deconvolution on simulated sparse LFM images instead of observed LFM images, the final resolution and deconvolution time was improved.

Data-driven methods infer the mapping relationship between the 3D sample volume and an LFM image from a dataset of image-volume pairs. This is usually done by utilizing deep neural networks (DNNs) due to their powerful non-linear fitting capacity. The PSF measurement information here is optional as long as the methods can implicitly learn the underlying physical process *H*^−1^ correctly. However DNNs require a large amount of training data, rely on numerous parameters, and usually require long training times. However, in return they offer higher reconstruction accuracy and real-time inference speed (Wagner et al., 2021). Wang Z. et al. (2021) first applied a variant U-Net DNN (Ronneberger et al., 2015) to a blind LFM deconvolution algorithm and achieved real-time zebrafish brain volumetric reconstruction (Figure 4B). Wagner et al. (2021) then introduced a hybrid light-sheet and LFM system they termed HyLFM, which used light-sheet microscopy data to supervise a DNN-based blind deconvolution algorithm for LFM images. This method improved blind deconvolution performance because both LFM and light-sheet data were captured with the same optics, and therefore implicitly used the same measurement matrix at the time of capture. While model-based LFM deconvolution algorithms are still in their infancy, future improvements will likely incorporate ideas from newly-emerging physics-informed machine learning techniques (Zhang and Ghanem, 2018; Shlezinger et al., 2019; Ongie et al., 2020; Khan et al., 2022; Yanny et al., 2022).

## 4. New developments: hybrid LFMs

New developments in recent years have combined LFM with various types of illumination and conventional optical microscopy systems. These novel hybrid systems aim to improve the performance of the system by offering better spatial resolution, signal to noise ratio, and contrast. Here we focus on methods that have been applied to zebrafish.

### 4.1. Confocal LFM

**Figure 5:**
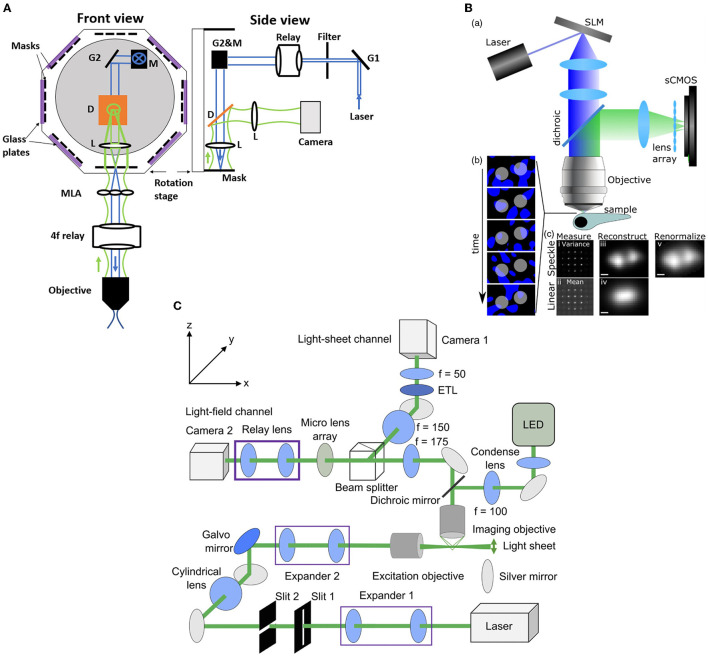
Hybrid LFM systems. **(A)** Confocal LFM (Zhang et al., 2021a), front and side views. G: scanning galvo mirror; Relay: achromatic relay lens system; M: mirror; D: dichroic mirror; L: achromatic lens. The mask is placed on a rotational stage. **(B)** Structured light LFM (Taylor et al., 2018). Top, speckle-based LFM utilizes controlled speckle illumination by imaging an SLM pattern with a random phase mask onto the objective back focal plane. Bottom left, fluorescent beads are illuminated with random speckle patterns displayed into the SLM and the resulting light is recorded. Bottom right, comparison of the speckle, top, and linear, bottom, LFM by taking the variance and the mean of the recorded data. The middle picture shows the differences in the PSF measurements. The beads are resolved in the speckle LFM while unresolved in the linear LFM. Scale bar 2 μm **(C)** Light-sheet LFM (Wang et al., 2019): Simultaneous imaging by light-sheet and light-field modalities. On the right, a zoomed-in view of the detection paths is shown. **(B)** reproduced with permission from Taylor et al. (2018), **(C)** reproduced with permission from Wang et al. (2019) ©The Optical Society.

Zhang et al. (2021a) introduced an improvement to the XLFM method which they termed confocal LFM (Figure 5A). This reduced the degradation of SNR due to out-of-focus light by shaping the excitation laser beam into a plane which passes through a slit. This xz plane was then scanned in the z direction, thus reducing out-of-focus light without discarding any fluorescence from the in-focus volume. Zhang et al. (2021a) demonstrated the performance of this system on both a movement-restrained zebrafish (volume imaging rate of 6 Hz) and a freely moving zebrafish, using a similar motion-cancelation system to Cong et al. (2017). This system improved the spatial resolution to 2 × 2 × 2.5 μ*m*^3^ over a cylindrical imaging volume of 800 μm diameter and 200 μm height. Moreover, this system eliminated the reconstruction artifacts near the focal plane presented in the conventional LFM (Zhang et al., 2021a).

### 4.2. Structured light LFM

Taylor et al. (2018) combined conventional LFM with speckle-based structured illumination to enhance spatial resolution (Figure 5B). Speckle illumination was introduced by placing a spatial light modulator (SLM) in the illumination path with a random phase mask, and imaging at the back focal plane of the objective. Using this neuronal activity was recorded in immobilized zebrafish at 10 Hz volume rate with suppressed background fluorescence and an increase in spatial resolution up to 1.5x compared to conventional LFM. Wang D. et al. (2021) used high contrast volumetric grating patterns to illuminate the live immobilized zebrafish using a digital micro-mirror device which was placed conjugate to the sample, which they termed structured illumination LFM (SI-LFM). Compared to conventional LFM, SI-LFM identified more neurons when imaging zebrafish, and had 3x improved SNR and 2x improved spatial resolution. However the FoV, 200 μ*m* by 250 μ*m*, was limited by the size of the micro-mirror device, which caused a trade-off with lateral resolution.

### 4.3. Light-sheet LFM

Combining the capability of LFM to capture volumetric images with the capability of light sheet excitation to optically section and scan the volume can lead to significant improvement in contrast and SNR. in immobilized zebrafish in a volume of 350 × 300 × 32 μ*m*^3^ and acquisition frame rate of 10 Hz using a combination of light sheet excitation and light field imaging, termed LSLFM (Figure 5C). Compared with conventional LFM, LSLFM produced 3.2x higher SNR and identified more active neurons. Wagner et al. (2021) used HyLFM with selective-plane illumination to achieve calcium imaging of immobilized zebrafish larvae over 350 × 280 × 120 μ*m*^3^ at 10 Hz. However these techniques require multiple measurements compared to conventional LFM, and thus trade off SNR for speed.

## 5. Conclusions

Brain imaging of moving zebrafish is a rapidly developing area with several exciting recent advances. However there are a number of limitations of existing techniques which will hopefully be addressed in future work.

- Current approaches are based on nuclear-targeted calcium indicators which have relatively slow temporal dynamics, with decay lasting several seconds (though see Zhang et al., 2023). This makes correlating behavior with neural activity particularly challenging, given that the timescale of sensorimotor integration during hunting events is at least an order of magnitude faster than this. This problem could potentially be addressed by the development of robust voltage indicators suitable for whole-brain imaging (Böhm et al., 2022).
- Current approaches (including LFM) are based on 1-photon imaging, which stimulates the fish's visual system and thus potentially interferes with the fish's ability to respond to visual stimuli such as prey items. This could be addressed by developing faster 2-photon volumetric imaging methods.
- Although current techniques approximate single-neuron resolution, this resolution is still significantly lower than that achieved by more conventional imaging techniques for head-fixed animals.
- In their natural environment zebrafish larvae make use of vertical movements through the water column, and in particular prefer to strike at prey items from below (Bolton et al., 2019; Mearns et al., 2020). In contrast current techniques require a water depth of barely more than the height of the fish, thus significantly restricting their normal range of movement and possibly causing stress. This could be addressed by image set-ups that allow very rapid vertical movements of the light field or focal plane.
- The techniques of Cong et al. (2017) and Kim et al. (2017) rely on immediate cancelation of the fish's movements so as to maintain it within the field of view. However Kim et al. (2017) found that enabling such cancellation caused substantial reductions in the average bout duration, maximum bout angle, and maximum bout speed and bout displacement. In addition some movements of the fish (for instance during the final stages of hunting) are too rapid to be exactly canceled using current techniques. These issues could be addressed by the development of methods for retaining the fish within the field of view both more rapidly and without relying on moving the dish containing the fish.

## Author contributions

All authors listed have made a substantial, direct, and intellectual contribution to the work and approved it for publication.
